# Targeting immunometabolism mediated by the IDO1 Pathway: A new mechanism of immune resistance in endometrial cancer

**DOI:** 10.3389/fimmu.2022.953115

**Published:** 2022-09-02

**Authors:** Anna Passarelli, Carmela Pisano, Sabrina Chiara Cecere, Marilena Di Napoli, Sabrina Rossetti, Rosa Tambaro, Jole Ventriglia, Federica Gherardi, Eva Iannacone, Sergio Setola Venanzio, Francesco Fiore, Michele Bartoletti, Giosuè Scognamiglio, Daniela Califano, Sandro Pignata

**Affiliations:** ^1^ Department of Urology and Gynecology, Istituto Nazionale Tumori Istituto di Ricovero e Cura a Carattere Scientifico Istituto di Ricovero e Cura a Carattere Scientifico (IRCCS) Fondazione G. Pascale, Naples, Italy; ^2^ Radiation Oncology Unit, Istituto Nazionale Tumori Istituto di Ricovero e Cura a Carattere Scientifico Istituto di Ricovero e Cura a Carattere Scientifico (IRCCS) Fondazione G. Pascale, Naples, Italy; ^3^ Radiology Unit, Istituto Nazionale Tumori IRCCS Fondazione G. Pascale, Naples, Italy; ^4^ Interventional Radiology Unit, Istituto Nazionale Tumori Istituto di Ricovero e Cura a Carattere Scientifico (IRCCS) Fondazione G. Pascale, Naples, Italy; ^5^ Medical Oncology and Cancer Prevention Unit, Department of Medical Oncology, Oncology Referral Center, Aviano, Italy; ^6^ Surgical Pathology Unit, Istituto Nazionale Tumori IRCCS Fondazione G. Pascale, Naples, Italy; ^7^ Functional Genomic Unit, Istituto Nazionale Tumori Istituto di Ricovero e Cura a Carattere Scientifico (IRCCS) Fondazione G. Pascale, Naples, Italy

**Keywords:** endometrial cancer, indolamine 2, 3-dioxygenase (IDO), tryptophan, kynurenine, immune metabolism, immune suppression, immunotherapy

## Abstract

Immunotherapy is acquiring a primary role in treating endometrial cancer (EC) with a relevant benefit for many patients. Regardless, patients progressing during immunotherapy or those who are resistant represent an unmet need. The mechanisms of immune resistance and escape need to be better investigated. Here, we review the major mechanisms of immune escape activated by the indolamine 2,3-dioxygenase 1 (IDO1) pathway in EC and focus on potential therapeutic strategies based on IDO1 signaling pathway control. IDO1 catalyzes the first rate-limiting step of the so-called “kynurenine (Kyn) pathway”, which converts the essential amino acid l-tryptophan into the immunosuppressive metabolite l-kynurenine. Functionally, IDO1 has played a pivotal role in cancer immune escape by catalyzing the initial step of the Kyn pathway. The overexpression of IDO1 is also associated with poor prognosis in EC. These findings can lead to advantages in immunotherapy-based approaches as a rationale for overcoming the immune escape. Indeed, besides immune checkpoints, other mechanisms, including the IDO enzymes, contribute to the EC progression due to the immunosuppression induced by the tumor milieu. On the other hand, the IDO1 enzyme has recently emerged as both a promising therapeutic target and an unfavorable prognostic biomarker. This evidence provides the basis for translational strategies of immune combination, whereas IDO1 expression would serve as a potential prognostic biomarker in metastatic EC.

## Introduction

Endometrial cancer (EC) is the most common gynecologic malignancy in Europe, and its prevalence is increasing. EC makes up 2% of all new cancer cases (1). It is typically detected in the early stages when the disease is confined to the uterus for most patients. The 5-year survival rates are high for patients with early-stage disease, and the 5-year survival rates of 76% have been reported for all patients with EC in Europe (all disease stages) (2).

In 2013, the Cancer Genome Atlas (TCGA), by evaluating the genomic and epigenomic landscapes of primary EC, delineated four distinct molecular subtypes, namely polymerase ϵ (POLE)-mutant/hypermutated, microsatellite instability-high (MSI-H), copy number low, and copy number high. This molecular classification reflects the underlying tumor biology and potential therapeutic strategies (3).

In this regard, EC cells and the tumor microenvironment (TME) have been shown to modulate the immune response. Firstly, EC cells possess the ability to activate programmed cell death protein 1 (PD-1) signaling, an immune checkpoint receptor able to downregulate the immune response by overexpressing programmed death-ligand 1 (PD-L1) and 2 (PD-L2). PD-L1 and PD-L2 bind PD-1 expressed on tumor-infiltrating CD4 and CD8 T cells, inactivating them in the TME (4). Immunohistochemical studies have described PD-1 and PD-L1 expression levels (40%–80% in endometrioid, 10%–68% in serous, and 23%–69% in clear cell subtypes, respectively) in EC, representing the highest expression within gynecologic cancers (3, 5). Secondly, EC subtypes with high tumor mutational burden (e.g., POLE-mutant/hypermutated and MSI-H) are highly immunogenic and exhibit more tumor-specific neoantigens, resulting in increased CD4 and CD8 tumor-infiltrating lymphocytes (TILs) and a compensatory upregulation of immune checkpoints (6). Increased TILs, an indicator of the anticancer immune response, have been associated with improved outcomes in EC (7).

This peculiar TME combination of increased mutational load, TILs, and PD-1/PD-L1 expression makes EC an ideal target for immunotherapeutic interventions. When considering therapeutic targets, it is important to note that EC was recently shown to have the highest prevalence of MSI across 30 human cancer types; approximately 30% of primary EC are MSI-H, and 13%–30% of recurrent ECs are MSI-H or DNA mismatch repair system defectives (dMMR) (8). This subgroup is characterized by low copy-number aberrations and a high mutational burden (8). Because the MSI-H status is a biomarker of response to immune checkpoint inhibition, in 2017, pembrolizumab (KEYTRUDA, Merck), an anti–PD-1 monoclonal antibody, was approved for the treatment of MSI-H or dMMR solid tumors, such as in EC (9). In April 2021, the FDA approved dostarlimab (Jemperli), an anti–PD-1 monoclonal antibody, to treat recurrent or advanced dMMR EC that has progressed on or following prior treatment with platinum chemotherapy (9).

Unfortunately, women with advanced and recurrent EC still have limited therapeutic options following standard therapy based on a platinum-based regimen (9). Given the critical role of the immune dysregulation process in EC progression, and considering that EC is more likely to benefit from immunotherapy than other gynecological malignant tumors, the use of immune checkpoint inhibitors has been explored as a therapeutic mechanism, both as monotherapy and in combination with targeted agents (5, 9). Most advanced EC patients are expected to receive immunotherapy alone or in combination, either in the first or the second line, worldwide. The treatment of patients resistant to immunotherapy is thus a clear unmet need. Therefore, the mechanisms of resistance to immunotherapy should be better investigated.

It has been suggested that the immunometabolic dysregulation mediated by the indolamine 2,3-dioxygenase 1 (IDO1) pathway protects EC cells from the cytotoxicity induced by T cells, thus actively generating an immunosuppressive milieu (10–12). The IDO1 enzymatic activity catalyzes the first rate-limiting step of the so-called “kynurenine (Kyn) pathway”. It depletes the tissue microenvironment of the essential amino acid l-tryptophan by converting it into the immunosuppressive metabolite l-kynurenine. In this review, we discuss the role and features of the TME in EC by focusing on the involvement of immunometabolism mechanisms mediated by the IDO1 pathway and its potential prognostic role. Finally, we also evaluate the potential application of targeting the IDO1 pathway in the therapeutic strategy of EC for overcoming immunotherapy resistance.

## IDO1 and immune functions

IDO1 is known to exert immune regulatory functions in several conditions, comprising infection, allergy, pregnancy, autoimmunity, chronic inflammation, transplantation, and mechanisms for the immune escape of tumors (13, 14). Investigation of other functions of IDO1, such as those related to its effect on vascular biology, nociception, and the central nervous system, is beyond the scope of this review and, therefore, will not be addressed.

### Mechanisms of the IDO1 immune function

In physiological conditions, IDO1 is expressed primarily in mucosal tissues, such as in the lung and placenta by endothelial cells, in the woman’s genital tract by epithelial cells, and also in lymphoid tissues by mature dendritic cells (DCs) with a phenotype (CD83^+^, DC-LAMPþ^+^, langerin^−^, CD123^−^, and CD163^−^) different from plasmacytoid DCs (15). The IDO1 activity is regulated by metabolic factors, such as heme cofactor, substrate supply, redox potential, and nitric oxide (NO). The inducible NO synthase (iNOS) enzyme is induced by interferon (IFN)-γ with subsequent NO production and blockage of IDO1 activity. Hence, IFN-γ co-induces iNOS and IDO1, but metabolic cross-regulation may override the IDO1-mediated one (16). The IDO1 pathway activity can also be reduced by lowering the IDO1 enzyme levels. In some cells, IDO1 levels are regulated by SOCS3, which sends IDO1 to proteasomal degradation (17, 18).

In some settings, the trade-off between tolerance and immunity seems to depend on factors altering the balance between local pro-inflammatory signals and the immunosuppressive activity by IDO1 (13). Because immune-related molecules induce *IDO1* gene expression, the IDO1 pathway also occurs during inflammation in many tissues, especially in case of sustained inflammation. Indeed, *IDO1* gene activation manifests together with the production of pro-inflammatory cytokines locally. IDO1 enzymes are intracellular, but their effects are not limited to the cells expressing IDO1 as they can act in a paracrine fashion. There are two main mechanisms through which IDO1 modulates immune responses: innate or inflammatory IFN-dominated responses, which cause a short but intense course of tryptophan degradation and Kyn metabolites’ production, and a transforming growth factor β (TGF-β)–driven self-maintaining form of intracellular signaling activity, which confers plasmacytoid DCs an immunosuppressive phenotype (14).

With regard to the IFN-mediated response, the IDO1 enzyme metabolizes the essential amino acid tryptophan producing soluble factors, such as Kyn and downstream metabolites, which strengthen an immunosuppressive milieu (10–12, 18). The production of secreted Kyn metabolites and the tryptophan-depleted environment can be sensed by neighboring cells (15). When Kyn pathway metabolites bind to the ligand-activated transcription factor aryl hydrocarbon receptor (AhR), it exerts immunosuppressive effects through the suppression of antitumor immune responses (19, 20), the promotion of the differentiation of FOXP3^+^ T_regs_ (regulatory T cells) (21, 22), and the decrease in the immunogenicity of DCs mediated by IDO1 expression (22). Tryptophan depletion is also a potent regulatory signal, as it activates molecular stress-response pathways, such as GCN2 kinase and mTOR (13). Although direct effects of IDO1 on the mTOR pathway have not been found yet, it is plausible that mTOR is a downstream pathway affected by IDO1 because amino acid withdrawal can affect this nutrient-sensing pathway (15, 23). Indeed, mTOR activity is inhibited during inflammatory responses where amino acids are catabolized by IDO1 and other enzymes (23–25). Regarding GCN2, it appears that the latter inhibits T effector cells while enhancing T_reg_ activity (15). GCN2 activation by IDO1 leads to cell-cycle arrest and functional anergy in CD8^+^ T cells (26). In contrast, in CD4^+^ T cells, it blocks T_H_17 differentiation (27, 28) and promotes *de novo* T_reg_ differentiation and activation of functional suppressor activity in mature T_regs_ (29, 30).

IFNs are the most important regulators of IDO1 expression. They activate the JAK/STAT complexes to induce transcription of many IFN-stimulated genes, such as the *IDO* genes. Mammalian *IDO1* gene promoters possess IFN-stimulated response elements and IFN-activated sites (16). Other factors capable of triggering IDO1 transcription comprise regulatory cytokines, such as IL-10 and TGF-ß, and AhR ligands such as Kyn (16). Although these inflammatory insults can induce IDO1 expression, multiple factors restrict its expression and regulate its activity (e.g., the IFN-induced IDO1 expression is regulated by the transcriptional factor DAP12) (17, 18).

In professional antigen-presenting cells (APCs), such as DCs, IDO1 can confer tolerogenic phenotypes by acting as a direct intracellular signaling molecule (13, 14). In the presence of IDO1 activity, APCs start producing inhibitory cytokines, such as TGF-β, instead of inflammatory cytokines (31–33). Whereas acute responses are best controlled by the IFN-γ–IDO axis, TGF-β is critical in establishing a regulatory, long-lasting phenotype in DCs (14). *In vitro* experiments showed that, in response to TGF-β, the *IDO1* promoter began to be transcriptionally active and maintained considerable activity later and for longer compared with that of IFN-γ. These effects remained sustained after TGF-β wash-up (14). Moreover, TGF-β–conditioned DCs and CD4^+^ T cells cultured with or without anti–TGF-β showed that the emergence, but not maintenance, of CD4^+^Foxp3^+^ T cells depended on TGF-β, suggesting that once induced, the regulatory population was not contingent on TGF-β. These elements suggest that TGF-β–dependent signaling in DCs induces IDO1 expression and a regulatory phenotype, which does not need TGF-β to be sustained (14).

These two mechanisms (IFN-induced and TGF-β–induced IDO1 expression) underline the importance of IDO1 upregulation in altering the whole local milieu from immunogenic to tolerogenic and changing the nature of the APC itself (14, 16). As stated before, IDO1 expression does not act exclusively on the cell expressing it but also in close-by cells, such as T cells interacting with APCs (15). This enables APCs to generate and sustain the function of T_regs_ through the combined effects of tryptophan starvation and Kyn acting *via* the AhR of T cells (14).

### Immunosuppression by IDO1 in the tumor microenvironment

**Figure 1:**
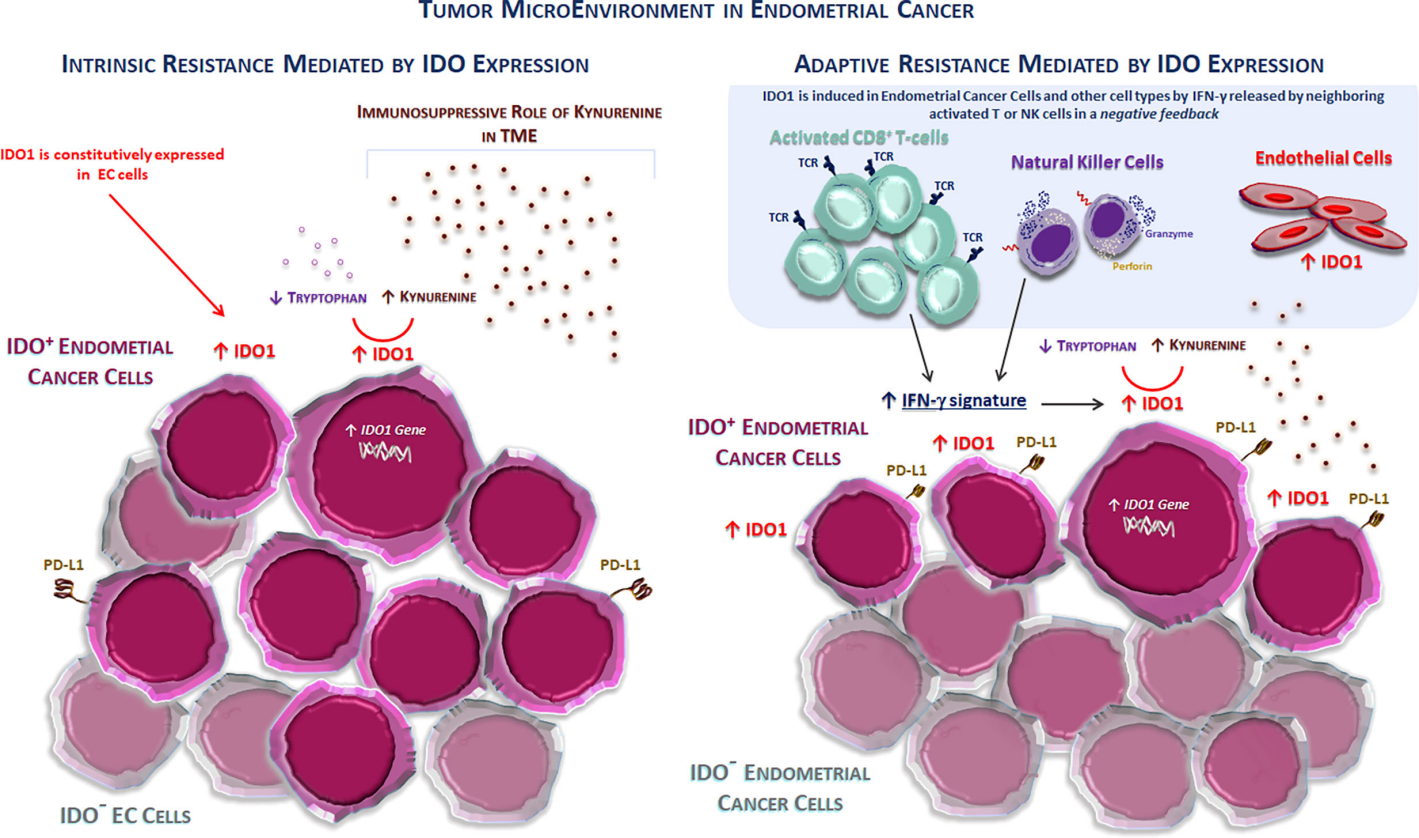
The tumor microenvironment in endometrial cancer. Two different mechanisms of immune resistance mediated by the IDO1 enzyme activity and expression in the TME in endometrial cancer. (Left) IDO1 is constitutively expressed in cancer cells (intrinsic or primary resistance) typically in non-inflamed tumors, such as endometrial and ovarian cancers. This expression prevents the accumulation of the activated antitumor CD8^+^ T cells, thus inducing an immunosuppressive TME. (Right) IDO1 is induced in endometrial cells (IDO + EC cells) and other cell types (stromal and endothelial cells) by IFN-γ released by neighboring activated T or NK cells in the context of a negative feedback loop (adaptive or acquired resistance). EC, endometrial cancer; IDO1, indolamine 2,3-dioxygenase 1; PD-L1, programmed death-ligand 1; TCR, T-cell receptor; TME, tumor microenvironment.

More than 60% of human tumors possess cells that express IDO1. These include tumor cells and stromal and endothelial cells in varying proportions according to tumor types. The gene expression data obtained from the TCGA database and immunolabeled samples show that the carcinomas of the cervix, followed by the endometrium, bladder, kidney, and lung, are the highest IDO1-expressing carcinomas. In particular, about 80% of EC expresses IDO1 (15). The expression of functional IDO1 in these tumors is constitutive, indicating that the *IDO1* gene is active regardless of environmental factors (15, 34). This constitutive IDO1 expression represents a critical mechanism of intrinsic/primary immune resistance, limiting both accumulation and proliferation of TILs and making these tumors “cold” (i.e., not triggering a strong immune response), as shown in **Figure 1**.

Moreover, like PD-L1, IDO expression seems more common in mismatch repair–deficient ECs than mismatch repair–intact tumors (not specified if only IDO1 or IDO2 as well) (35). Interestingly, in a study by Liu et al. (12), the percentage of primary (38%) and metastatic (43%) EC samples expressing IDO (not specified if only IDO1 or IDO2 as well) was significantly lower when compared with normal endometrium samples (57%). On the other hand, recurrent EC specimens showed a higher percentage than normal endometrial samples. Despite this, blocking the IDO1 pathway might be a useful treatment option in some settings, given that IDO is highly expressed in 21% of primary EC (12). In the same study, IDO was found to be expressed not only in the cytoplasm but also apically, and cells expressing IDO were in close proximity to the tumor vessels (12). Thus, tumoral IDO expression, as well as that of PD-L1, tends to be directed to the infiltrating edge of endometrial carcinomas suggesting an ongoing adaptive immune response (12, 35).

Stromal expression of IDO1 is usually observed in tumors rich in immune infiltrates, such as TILs. Because IDO1 transcription is strongly induced by IFN-γ, the IDO1 expression in inflamed TME likely results from IFN-γ produced by TILs. Consistently, the transcriptomic analysis reported a strong correlation between CD8^+^ T-cell infiltration and IDO1 expression in tumor models, such as melanoma. This is similar to *CD274* (the gene encoding PD-L1), which is also inducible by IFN-γ, whose expression is also correlated with TILs. This represents a typical mechanism of adaptive resistance, where the immune system recognizes cancer, but protects itself by adapting to the immune attack mediated by infiltrating T cells through the production of immunosuppressive factors, such as PD-L1, TGF-ß, and IDO1 (13). Other mechanisms activated by tumor cells to escape the immune surveillance include the paracrine production of negative mediators, such as adenosine, VEGF, and overexpression of inhibitory immune checkpoints. The intrinsic or adaptive resistance mechanism mediated by IDO1 expression in the TME is shown in **Figure 1**.

Patients with EC have increased tryptophan degradation resulting in higher serum Kyn concentrations and a higher Kyn/tryptophan ratio compared with healthy woman controls (36). Tryptophan depletion through IDO1 overexpression favors cell-cycle arrest and apoptosis in T lymphocyte or NK cells (10, 11). Moreover, in NK cells, Kyn downregulates the specific triggering receptors NKp46 and NKG2D, suppressing the killer functions (11). Given that the TME is depleted of tryptophan, it can be expected that immune cells and cancer cells will suffer from tryptophan shortage as they are found close to each other. Cancer cells may be less sensitive to this condition than T or NK cells, resulting in localized tolerance within the TME and the contribution to the tumor escape from host immune surveillance (11). In fact, IDO1 induces a novel tryptophan transporter expression in mouse and human cancer cells (37). Such alternative means of tryptophan intake are probably involved in maintaining an adequate cellular tryptophan status when the microenvironment becomes depleted (38).

Because IDO1 expression is found in tumor cells of different types of cancers, many studies report that a high IDO1 expression is associated with a negative effect on prognosis (11, 13, 16, 39–45). Tumors in this category include EC, colon cancer, melanoma, ovarian cancer, brain tumors, acute myelogenous leukemia, and others (16). The prognostic significance of intra-tumoral IDO expression has been investigated in large cohorts of EC patients. Indeed, the IDO expression in EC correlates with the frequency of nodal metastases and lower numbers of intra-tumoral CD8^+^ T lymphocytes (not specified if IDO1 or IDO2 as well) (38, 43, 46). The intra-tumoral high IDO expression has a negative impact on survival in advanced EC. Finally, the IDO expression in EC could be an independent prognostic factor for impaired progression-free survival (35) and independently associated with poor disease-specific survival in a general cohort of EC patients and among patients with early-stage EC, but not in subgroups with advanced stage and an endometrioid tumor type (38).

## Immune checkpoint inhibitors in endometrial cancer

Treatments based on immune checkpoint blockade (ICB), especially PD-1 or PD-L1 inhibitors, have been explored as a therapeutic strategy in advanced EC, both as monotherapy and in combination with cytotoxic chemotherapy, other immunotherapy, or targeted agents. In microsatellite stable (MSS) or PD-L1–positive advanced EC, response rates ranging from 3% to 23% have been observed with PD-1 inhibitors, such as nivolumab and dostarlimab, and with PD-L1 inhibitors, such as atezolizumab, avelumab, and durvalumab (5). In MSI-H or dMMR-advanced ECs, PD-L1 inhibitors, such as durvalumab and avelumab, have shown response rates of 43% and 27%, respectively. Conversely, the PD-1 inhibitors, such as dostarlimab and pembrolizumab, appear more effective, showing response rates of 49% and 57%, respectively (5). However, the tumoral expression of PD-1 and PD-L1 is just one of the many potential mechanisms of immune evasion in EC.

Lenvatinib is a selective inhibitor of VEGF-α, KIT, and RET and is a potent angiogenesis inhibitor. It has also been shown to be an effective immunomodulator. Lenvatinib decreases tumor-associated macrophages, increases T-cell population, upregulates the type I IFN signaling pathway, and leads to the activation of CD8^+^ T cells. In 2019, the FDA granted accelerated approval for the combination therapy of lenvatinib and pembrolizumab for the treatment of advanced non–MSI-H and non-dMMR EC that has progressed following prior therapy, according to substantial activity in phase Ib/II KEYNOTE-146/Study 111 (5). Later, in a randomized phase III trial (KEYNOTE-775/Study 309), lenvatinib plus pembrolizumab led to significantly longer progression-free survival and overall survival than chemotherapy among patients with advanced EC who had received one or two previous platinum-based chemotherapy regimens (47). To verify whether pembrolizumab plus lenvatinib is superior to chemotherapy in terms of progression-free survival and overall survival in patients with mismatch repair-proficient tumors and all patients even in the first line, the ENGOT-en9/LEAP-001 trial is currently ongoing (48). This trial has the potential to define the new standard of first-line treatment in advanced EC.

## Targeting IDO1 pathway in cancer

### Efficacy of IDO1 blockade

IDO1 inhibition has already been shown to be effective in preclinical settings. Over the past decade, intense efforts have been made to develop IDO1 inhibitors, and several small-molecule IDO1 inhibitors have been reported.

Among the several investigations with murine models attempting the clinical application of the IDO1 inhibitor therapy, the synthetic analog of tryptophan, 1-methyltryptophan (1-MT), best known as indoximod, is by far the most employed IDO1 inhibitor in the preclinical literature (49). 1-MT was first described as a competitive inhibitor of the IDO1 enzyme in the early 1990s. However, unlike its L-isomer, which has shown weak inhibitory activity, the D-1-MT isomer neither binds nor inhibits the purified IDO1 enzyme. In contrast to direct enzymatic inhibition of IDO1, indoximod acts downstream of IDO1 to stimulate mTORC1, which is a central regulator for cell growth (49). 1-MT, while not cytotoxic itself, may heighten the cytotoxic effects of chemotherapeutic agents in IDO1-expressing tumors (11). In murine P815 mastocytoma, tumor growth was significantly reduced by 1-MT in immunized mice, whereas, in mice depleted of T cells, the 1-MT effect was abolished (11). IDO1 inhibition has shown to increase the therapeutic efficacy of checkpoint inhibitors, cancer vaccines, or even chemotherapy (34, 50–52) in mice and human tumors grafted into immunodeficient mice reconstituted with human lymphocytes (53). This background indicates that murine and human lymphocytes are sensitive to IDO1-mediated immune suppression. Clinical trials with 1-MT have been conducted in different clinical settings, albeit none in EC. Results indicated that when used in monotherapy, indoximod exerted limited anticancer efficacy. In contrast, the combination of indoximod with other therapies including cancer vaccines, immune checkpoint inhibitors, and chemotherapy showed an antitumor efficacy (49).

Of the various IDO1 inhibitors that entered clinical trials targeting different advanced solid tumors, those in combination with the anti–PD-1 antibody have displayed better cooperativity, which would help overcome the drug resistance and maximize the survival benefits of patients (54). In the development of IDO1 inhibition targeting, the most advanced is epacadostat, which has already been tested in several clinical trials. Epacadostat is an orally available, highly specific, reversible competitive IDO1 inhibitor with over 1,000-fold selectivity to IDO1 over IDO2 or tryptophan dioxygenase. *In vitro* and *in vivo* studies showed that epacadostat reduced the tumor growth and promoted the proliferation of both T and NK cells. Preclinical studies showed that epacadostat and immune checkpoint inhibitors had a synergy effect, and several clinical trials were initiated to evaluate the combination of epacadostat with immune checkpoint inhibitors.

Based on encouraging clinical results in early-phase trials in other tumors such as melanoma, a crucial randomized phase III study (ECHO-301/KN-252; NCT02752074) was launched to test the benefit of adding epacadostat to pembrolizumab therapy in the first line. This study investigated the efficacy, safety, and tolerability by combining pembrolizumab with epacadostat or placebo in patients with unresectable or metastatic melanoma. Unfortunately, the negative results hampered the development of IDO1 inhibitors (55). Therefore, several phase III trials were terminated and withdrawn. Among these clinical trials, the study NCT03310567, designed for patients with recurrent or metastatic EC, was terminated due to these reasons and low enrollment (**Table 1**).

**Table 1:** Current development status of IDO1 inhibitor strategies in advanced endometrial cancer.

Clinical trial	Drug	Mechanism of action	Pharmaceutical company	Phase of development	Condition or disease	Drugs combination	Status
**NCT03310567**	Epacadostat	IDO1 inhibitor	Incyte Corporation, Merck Sharp & Dohme	II	Recurrent/metastatic endometrial carcinoma	Epacadostat; pembrolizumab	Withdrawn (sponsors pulled out of the study)
**NCT02178722**	Epacadostat	IDO1 inhibitor	Incyte Corporation, Merck Sharp & Dohme	I/II	Advanced selected cancers	Epacadostat; pembrolizumab	Completed
**NCT04106414**	BMS-986205	IDO1 inhibitor	Bristol-Myers Squibb	II	Endometrial cancer or endometrial carcinosarcoma	BMS-986205; nivolumab	Active, not recruiting
**NCT04463771 (POD1UM-204)**	Epacadostat	IDO1 inhibitor	Incyte Corporation	II	Metastatic endometrial cancer that has progressed on or after platinum-based chemotherapy	Epacadostat; INCMGA00012; pemigatinib	Recruiting

BMS-986205, linrodostat; INCMGA00012, retifanlimab; pemigatinib, FGFR 1,2,3 inhibitor.

The failure of the phase III trial with epacadostat in first-line metastatic melanoma is a key turning point in developing IDO1-targeting drugs (54). The mechanism of IDO1 inhibition and rational trial design should be a priority for discovering and developing an IDO1-targeting molecule. Interestingly, Van den Eynde et al. proposed several potential reasons for the negative outcome of using epacadostat plus immunotherapy in metastatic melanoma (56), such as insufficient IDO1 inhibition by epacadostat in the tumor; no selection of patients for tumoral IDO1 expression; no selection for patients refractory to immunotherapy; the adaptivity of the IDO1 expression mechanism in melanoma; compensatory expression of tryptophan dioxygenase or IDO2; the activation of the AhR by epacadostat, which drives immune suppression; and the insufficient blockade of the tryptophan–Kyn–AhR pathway by IDO1 inhibitors (56). Thus, it is urgent to address now the reasons for the clinical failure of epacadostat and whether IDO1 remains a critical immuno-oncology target. This can help determine the path forward in the clinical development of IDO1 inhibitors for cancer therapy.

### Rationale for the IDO1 blockade in endometrial cancer

**Figure 2:**
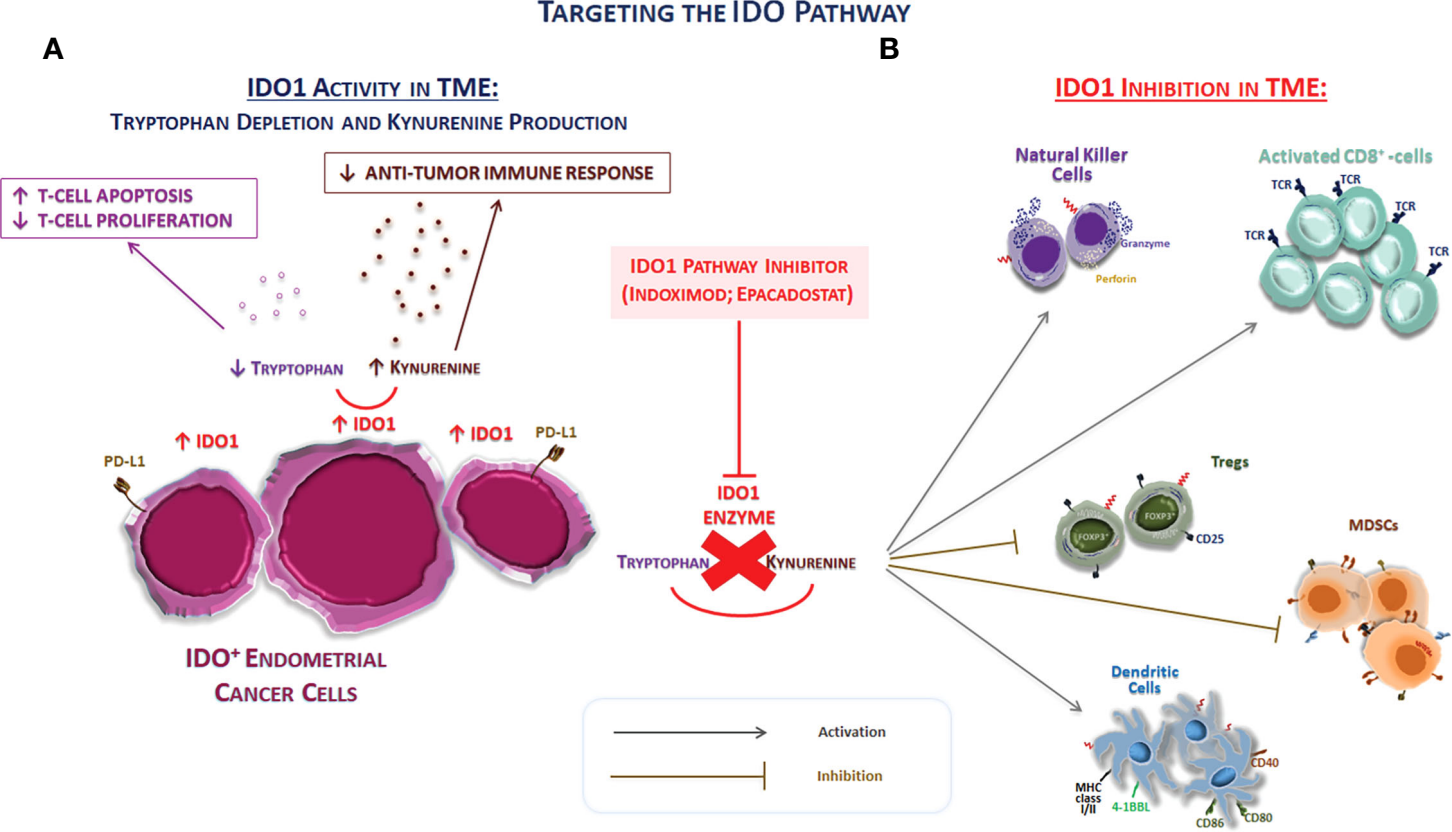
Targeting the IDO pathway. **(A)** The schematic representation of the effect of IDO1 on immune system cells of TME. IDO1 inhibits immune responses through several mechanisms, including the depletion of the essential amino acid tryptophan and the overproduction of kynurenine. The tryptophan depletion can inhibit T-cell proliferation arresting the cell progression cycle. In addition to the depletion of tryptophan, the accumulation of kynurenine exerts also immunosuppressive effects through the promotion of the differentiation of FOXP3^+^ T_regs_, the decrease in the immunogenicity of DCs, and the inhibition of T effector cell. Thus, IDO1 represents a driver of tumor-mediated suppression. **(B)** The IDO1 pathway inhibition directly acts on the modulation of both innate and adaptive immune system in TME. Thus, the IDO1 inhibition can potentially turn these “cold” tumors into “hot” tumors. Abbreviations: IDO1, indolamine 2,3-dioxygenase 1; MHC, major histocompatibility complex; MDSC, myeloid-derived suppressor cells; TCR, T-cell receptor; T_regs_, regulatory T cells.

To date, there are few data on how the concurrent presence of other anti-immune defenses may interfere with the effectiveness of anti–PD-1/PD-L1 therapies in solid cancers, including EC (12, 57). If the goal of immunotherapy is to activate T cells within the TME, the activation of immunosuppressive pathways, such as that of IDO1 by the same activated cells, must be avoided in order to not reduce nor inhibit the expected antitumor effect (**Figure 2**). Preclinical studies demonstrated this concern with IL-12 therapy (58) and adoptive cell therapy using CAR-T cells (59), whereas confirmation in the clinical setting is still lacking. IDO1 expression can reduce the effectiveness of immunotherapy regimens by turning tumor-associated cytotoxic T cells dysfunctional. Thus, immunotherapies inducing extensive inflammation at the tumor site might benefit from a combination with an IDO1 inhibitor medication, as exposing a tumor to immune recognition is of little utility if the immune system cannot effectively eliminate it (12, 16, 57). The IDO1 expression in EC spans the four molecular subtypes, with higher levels in dMMR tumors, particularly Lynch syndrome–associated EC, and the POLE subtype (35, 60–63). However, it remains common in mismatch repair–intact tumors, a group for which immunotherapy is not currently considered a viable option. Therefore, IDO1 targeting can be also effective in tumors without abnormalities in the mismatch repair system, although further research is warranted (35).

In light of these considerations, the blockage of the IDO1 pathway in ECs appears as an attractive treatment option, as IDO1 provides a direct mechanism of tumor protection against attack by closely located or contacting T lymphocytes (14). The high expression frequency of both IDO1 and PD-L1 in EC suggests that therapies targeting only the PD-1/PD-L1 axis may be turned down in this tumor type due to IDO1 interference with immune cell function (**Figure 2**). Moreover, almost all tumors expressing PD-L1 coexpress IDO (not specified if IDO1 or IDO2 as well), but more than half of tumor-expressing IDO lacks PD-L1 expression, suggesting that IDO-expressing tumors are significantly more common than PD-L1–expressing ones (35). Thus, combination therapy might be of clinical utility in this scenario.

### Clinical trials in endometrial cancer

**Table 1** gives an overview of the clinical investigations targeting the IDO1 pathway in advanced EC treatment. Of the four trials in EC, three investigated the use of epacadostat and one investigated the use of BMS-986205.

The first positive preliminary data from a phase I/II study (NCT02178722) of the combination of epacadostat with pembrolizumab in patients with selected advanced cancers such as endometrial adenocarcinoma showed that epacadostat with pembrolizumab was generally well tolerated, and efficacy data suggest promising clinical activity (64). In this study, seven patients (11%) had endometrial adenocarcinoma. Of these patients, one achieved complete response and one partial response (65).

NCT04106414 is a Memorial Sloan–Kettering Cancer Center investigator-initiated, single-center, randomized, open-label, phase II study to evaluate the activity of the PD-1 inhibitor nivolumab with and without the IDO inhibitor BMS-986205 (linrodostat) in patients with recurrent or persistent EC or endometrial carcinosarcoma and is currently in the recruiting phase.

Finally, POD1UM-204 (NCT04463771) is the most attractive ongoing trial. This is an umbrella study of PD-1 inhibitors (retifanlimab, INCMGA00012) alone or in combination with other therapies in patients with advanced EC who have progressed on or after platinum-based chemotherapy. Previously, the POD1UM-101 study provided encouraging efficacy data (cohorts A and B). Retifanlimab was well tolerated and demonstrated antitumor activity in patients with pretreated recurrent MSI-H or dMMR EC, consistent with other PD-1 therapies (66). In POD1UM-204, patients with advanced EC with disease progression on or after >1 platinum-based regimen are enrolled in four treatment groups based on prior immunotherapy exposure and tumor characteristics, such as MSH-I, dMMR, ultra-mutated POLE, and *FGFR* mutation. Indeed, approximately 16%–20% of advanced EC patients have *FGFR* mutation associated with more aggressive disease and significantly shorter progression-free survival and overall survival. Therefore, an additional clinical benefit could be expected from the PD-1 inhibitor in addition to the FGFR inhibitor (pemigatinib). Interestingly, group C provides the use of retifanlimab plus epacadostat in select participants who are allowed on prior checkpoint inhibitors. Conversely, group E provides the same combination therapy as retifanlimab plus epacadostat in patients naïve to checkpoint inhibitors.

Despite the relevant progress made so far, there are still some issues. It is unknown if the prolonged activation of AhR affects cancer progression, considering that its activation by IDO1 inhibitors may induce pro-carcinogenic effects and can be associated with poor prognosis. The reduced mTOR activity due to tryptophan depletion can be reactivated by tryptophan-mimicking IDO1 inhibitors that can act as fake nutritional signals, in turn causing artificial antitumor efficacy of these inhibitors. Indeed, mTOR can reactivate the T-cell function, thus overcoming the tumor immune escape (54). IDO1 is also known for participating in different aspects of vascular biology. In this regard, Kyn contributes to vasodilatation, acting as a vascular relaxing factor. Thus, the vascular-related side effects of drug IDO1 inhibition are possible (67). Finally, a more accurate clinical trial design, possibly through the stratification according to the IDO expression level, can also help overcome the risk of failure in EC model clinical studies.

## Conclusion

Advanced EC remains the most aggressive and life-threatening gynecologic malignancy, albeit remarkable therapeutic advances have been achieved. In several tumor types, including ECs and ovarian cancers, IDO1 expression can be observed in non-inflamed tumors and is confined to tumor cells themselves. Interestingly, this constitutive expression represents a mechanism of intrinsic immune resistance, which can prevent the accumulation of TILs in the TME, thus making these tumors “cold”. Given the presence of immune dysregulation in EC, the ICB has been explored as a therapeutic mechanism, both as monotherapy and in combination with cytotoxic chemotherapy, other immunotherapy, or targeted agents. IDO1-related ICB could potentially turn these “cold” tumors into “hot” tumors. A better understanding of the biological and molecular mechanisms involved in endometrial tumor progression and immune evasion is required. Overexpression of IDO1 by human EC cells is known to enhance tumor progression *in vivo*, and IDO1 inhibitors improve tumor rejection in mice models when combined with checkpoint inhibitors. Based on encouraging multiple preclinical models and results in early-phase trials, some randomized studies are ongoing also in advanced EC to test the benefit of adding IDO1 inhibitors to conventional immunotherapy. Immunotherapy combinations are relevant strategies aimed at restoring anticancer immunity and restraining primary and acquired resistance to immune checkpoint inhibitors.

In conclusion, based on these findings, clinical studies aiming at translating IDO1 inhibition strategies into EC treatment are required to evaluate the targeted blockade of IDO1 signaling as an additional, alternative, and effective future approach.

## Author contributions

All authors listed have made a substantial, direct, and intellectual contribution to the work and approved it for publication.

## Funding

This work was supported by the Ricerca Corrente RC 2022/2024, Project—”Theory enhancing”: “Valutazione dell’espressione dell’enzima indoleamina 2,3-diossigenasi-1 (IDO1) in associazione ad altri biomarcatori tissutali, genomici e bioumorali quali fattori prognostici e predittivi di risposta al trattamento immunoterapico con anticorpi monoclonali anti–PD-1 in pazienti con carcinoma endometriale ricorrente o avanzato con deficit di riparazione del mismatch del DNA (MMRd) o elevata instabilità dei microsatelliti (MSI-H)”.

## Acknowledgments

The authors wish to acknowledge Angela Maria Trujillo, Gelsomina Iovane, and Margherita Tambaro for the research assistance; the medical writer Fabio Perversi (Polistudium srl, Milan, Italy) for his help in drafting the manuscript; and Aashni Shah and Valentina Attanasio (Polistudium srl, Milan, Italy) for English and editorial assistance.

## Conflict of interest

SP received honoraria from MSD, Pfizer, AZ, Roche, Clovis, and GSK.

The remaining authors declare that the research was conducted in the absence of any commercial or financial relationships that could be construed as a potential conflict of interest.

## Publisher’s note

All claims expressed in this article are solely those of the authors and do not necessarily represent those of their affiliated organizations, or those of the publisher, the editors and the reviewers. Any product that may be evaluated in this article, or claim that may be made by its manufacturer, is not guaranteed or endorsed by the publisher.
